# Artificial womb technology and the significance of birth: why gestatelings are not newborns (or fetuses)

**DOI:** 10.1136/medethics-2019-105723

**Published:** 2019-08-31

**Authors:** Elizabeth Chloe Romanis

**Affiliations:** Centre for Social Ethics and Policy, University of Manchester Manchester School of Law, Manchester, UK

**Keywords:** embryos and fetuses, moral status, ethics, reproductive medicine, newborns and minors

## Abstract

In a recent publication, I argued that there is a conceptual difference between artificial womb (AW) technology, capable of facilitating gestation ex utero, and neonatal intensive care, providing incubation to neonates born prematurely. One of the reasons I provided for this distinction was that the subjects of each process are different entities. The subject of the process of gestation ex utero is a unique human entity: a ‘gestateling’, rather than a fetus or a newborn preterm neonate. Nick Colgrove wrote a response to my paper, claiming that my distinction between the subject of an AW and a newborn (in intensive care) was false. He claims that I have not accounted for the proper definition of ‘birth’ and that gestatelings are not a distinct product of human reproduction. Further, Colgrove posits that even if I can successfully distinguish gestatelings from preterms, such a distinction is morally irrelevant because the entities would have the same moral status. In this paper, I address the three challenges raised and defend the claim that gestatelings are unique entities. Moreover, I argue that moral status should not be considered ipso facto determinative in the debate about AWs.

In an earlier publication, I argued that there is a conceptual difference between partial ectogenesis (PE)^1^ (facilitated by artificial wombs (AWs)) and neonatal intensive care (NIC).^1^ I advanced several reasons for this distinction, one being that the subjects of each technology were different entities. The subject of PE, which I termed the ‘gestateling’,^1^ is undergoing the process of gestation, whereas the subject of NIC, a preterm neonate, is being assisted by incubation. The gestateling is a unique human entity, functionally distinct from the fetus and the newborn. Moreover, referring to the subject of an AW with unique terminology provides clarity in the discussion. Colgrove responded to my paper,^2^ arguing that gestatelings are not distinct from newborns. Colgrove claims I am over-reliant on medical definitions to distinguish gestatelings from fetuses, but do not adequately account for relevant definitions of birth that demonstrate gestatelings are equivalent to newborns. He claims that gestatelings are not a distinct product of human reproduction because they are newborns by definition. Finally, he suggests that any distinction between gestatelings and preterms would be morally irrelevant.^2^ Perhaps he was correct to identify that some of my claims could have benefited from further defence. This is not the same, however, as having proven, as he claims, that the distinction as I advanced it is incapable of surviving scrutiny.^2^ ^2^

## Birth

Colgrove argues that gestatelings just *are* newborn (by definition).^2^ He substantiates this only by citing the WHO’s definition of ‘live birth’.^3^ ^3^ He fails to acknowledge, however, that this definition delineates two events encompassed in the process of *complete* birth: first, the expulsion of the entity from a pregnant person, and second, the emergence of that entity from the process of gestation.^4^ Greasley explains that the birth process involves the developing human entity undergoing meaningful changes beyond changing location.^5^ These are biological adaptations enabling the entity to survive in an ex utero environment, for example, the clearing of fluid from the lungs to allow breath and the activation of the digestive system.^5^ Usually, these two events coincide: a baby is delivered by/from a pregnant person, and simultaneously, it makes the necessary adaptations for independent life. There has been little thorough examination of the process of birth^4^ because it is usually a straightforward uniform process.^4^ Both the change in location (facilitated by the pregnant person’s delivery) and the making of the necessary biological adaptations for life (facilitated by the newborn itself) have traditionally been thought of as coetaneous, with the same temporal boundaries. PE, however, demonstrates that these two occurrences are not coextensive; they are independent processes that merely *happen* to be naturally synchronised.

I previously described AWs as treating the gestateling ‘as if it had not been born’.^1^ Colgrove answered that being ‘a newborn means having been born recently’ and merely dismissed the nuanced features (such as behaviour) I suggested were material to a birth as irrelevant.^2^ He, therefore, does not answer the point. AWs continue gestation^5^, 6 so the gestateling does not complete the biological state changes in birth. Birth is not *completed*. Colgrove misconstrues birth as only an ex utero existence and a matter of semantics. This idea that ‘unborn’ is a strict quantitative concept measured on one binary is misleading. It is a reductive approach to a biologically complex situation involving multiple entities, the environment and their interaction. The gestateling undergoing PE is born only in a geographical sense. It should still be described as unborn because it has not completed *all* of birth.

Colgrove considers the case of complete ectogenesis (CE).^6^ He claims that the formal principle of justice^7^ requires that all subjects of AWs must be treated the same regardless of whether they are gestated in an AW from conception or after a partial pregnancy.^2^ He posits that the subject of PE is born and shares the same moral status as a newborn, and thus, we must also accept that the subject of CE has this same status.^2^ This seems implausible. If entitlement to equal treatment comes only from being biologically alive and ex utero, this logic counterintuitively suggests that a non-implanted embryo alive in vivo would also be ‘born.’ He fails to provide any substantive reasons why gestatelings should be considered *completely born*. In fact, he concedes that the subject of CE would, in a sense, be ‘unborn.’ His argument about equal treatment, therefore, works just as easily the other way, supporting my claim that even the gestateling that has been removed from a person’s uterus is different from a newborn. Alghrani and Brazier,^8^ Sander-Staudt,^9^ Gelfand and Shook,^7^ and Steiger^10^ all argue that the subject of CE should be considered born only at the point of removal from the AW, when emerging from gestation.^8^ Intuitively, the subject of CE is not born, and thus, if all subjects of the technology should be treated the same, the subject of PE is not born either.

## The exercise of independent life

Gestatelings and newborns are distinct because a gestateling exercises no independent capacity for life, whereas newborns shoulder the primary burden of sustaining themselves.^1^[7] This is an important distinguishing feature that Colgrave did not address in his paper. In English law,^8^ breathing, including assisted breathing, has been the focus of determining independent life in the law.[9]^11–13^ Breathing after birth is observable without sophisticated technology^4^ and demonstrates an obvious capacity for self-sufficiency. The newborn, with or without ventilator assistance, is compelled to breathe. The gestateling, however, does not use its lungs to acquire oxygen^14^ and thus has not made the most obvious biological adaptation (clearing the lungs to allow them to inflate^5^) for independent life. Even if one is persuaded that there are features other than breathing / tolerating artificial ventilation that might demonstrate the exercise of independent life 2 10 I argue that there are two qualities to sufficient proof that a human entity is engaged in the exercise of independent life, and the gestateling does not perform any activities of this nature.

First, activities sufficient to demonstrate the active exercise of independent life include only those that are suited to, and involve interaction with, the external environment. The significant feature of all the biological adaptions in birth is that they enable entities to survive in the external environment. Unlike a newborn, a gestateling remains dependent on a process of creation in a temporary environment. Greasley also highlights the importance of newborns also being responsive to environmental stimuli and interacting with other human beings as behavioural evidence of a meaningful completed birth.^5^ The gestateling is encased in the AW and is incapable of experiencing physical human interaction.^1^ This is a significant difference that affects how persons will perceive and respond to it.^11^

Second, activities demonstrating the exercise of independent life include only those that are exertive. The primitive signs of life (other than breathing) that Colgrove mentions in the definition of birth, such as a heartbeat, are evident in a foetus and while Colgrove could attempt to claim they are ‘active’, they demonstrate no self-sufficiency. It seems absurd to treat the primitive signs of life during gestation as evidence of self-sufficiency. We would not claim that a fetus sustained by a pregnant person was demonstrating ‘self-sufficiency’. The coordination of all bodily functions during gestation is always reliant on the gestational carrier (pregnant person or machine). There is a useful contrast to be made between living human tissue and an organically integrated live human entity. Embryos are created by the fusion of living tissue, and following brain death, organs remain sufficiently live for harvesting for transplantation.^12^ It seems hardly intuitive to consider these tissues ‘actively alive’.

## Moral status

Colgrove observes that I did indeed leave open the possibility that gestatelings and newborns have different moral statuses. However, he claims that any distinction between gestatelings and newborns is not morally relevant because they would have the same moral status.^2^ Colgrove does not substantiate this claim, focusing instead on attempting to establish that a gestateling is not a unique entity. I have demonstrated that there are some morally relevant differences between gestatelings and newborns. I will not further address this claim about any equivalence (or not) in moral status; rather I want to highlight that considering the moral status of the gestateling in this context is not useful in terms of isolating and addressing the important ethico-legal questions stemming from AWs.

There is generally a dichotomy in the literature between two alternative accounts of moral status. Some believe all human life is intrinsically valuable,^15^ others believe only the lives of persons (and, thus, not developing human entities) have intrinsic value.^16^ ^13^ There is little hope of unilaterally resolving the question of the moral worth of developing human entities.^14^ There are, however, practical problems with AWs that require resolution, such as how to select research participants for innovative technology or when an AW could be ‘switched off’.^17^ A way of conceptualising of the gestateling should be adopted that allows us to answer some of these emerging questions without falling into the trap of over-relying on theories of what the ‘fetus’ or ‘newborn’^15^ is and assumptions about a moral status attached to them. Moral status is limited as a consideration because it is subjectively attributed rather than innate. Furthermore, assigning a moral status does not in itself immediately tell us how entities should be treated, because once the status is assigned we must then make moral judgements about whether that status justifies certain treatment. This is evident from Colgrove’s paper, in which despite his conclusions that a gestateling is morally equivalent to a newborn, he concludes this ‘does not imply they have a right to life nor does it imply that have a right not to be killed.’^2^

Importantly, attempting to reduce the debate about AWs to a question of moral status frames AWs as something that only concerns the developing human entity. However, AWs are not just about gestatelings. The location of a developing human entity matters because when it is located inside the womb of a pregnant person, this impacts significantly on that individual. There can be no uncertainty about the need to respect the subjective preferences of the pregnant person and to allow them to make decisions about their body and gestational labour. Focusing entirely on the value of the fetus/gestateling neglects this fact that decisions about gestation impact on a pregnant person’s self. Choosing to opt for an AW or not, and in what circumstances, is still a decision that a woman^16^ should be entitled to make, and this decision should be framed with them, rather than the fetus/gestateling, at the centre.

## Footnotes

The process of gestation continued ex utero in artificial conditions (not dependent on a pregnant person).

It is essential to highlight that no challenge was raised to the claim that gestatelings are different entities from fetuses. Fetuses and gestatelings are different because one is dependent on a pregnant person and the other is not. This is interesting because perhaps a stronger challenge to my work can be made arguing that gestatelings and fetuses are not distinguishable, rather than that gestatelings and newborns are not distinguishable. This is because gestatelings are more ontologically similar to the fetus in utero in terms of behaviour, dependence on their environment, how they are interacted with and potentially in appearance than to ‘newborn babies’.

The definition of live birth given by the WHO is ‘…the complete expulsion or extraction from its mother of a product of conception, irrespective of the duration of the pregnancy, which after such separation, breathes or shows any other evidence of life….’^3^

In instances where it is not a straightforward or uniform process, it is usually considered a stillbirth, in that the entity either died as a fetus in utero, died during the process of being delivered or died almost immediately after being removed from the uterus because the entity was incapable of making the necessary biological adaptations to survive after gestation.

The authors of a 2019 pilot study of a refined AW system named ‘EVE therapy’ expressly state that the ‘central principle underling the iterative development of (EVE therapy)… is to treat extremely preterm infants as fetuses, rather than as small babies, and to avoid the use of pulmonary gas exchange’.^6^

Colgrove notes that these claims were beyond the scope of my original paper. I deliberately chose not to address matters of CE because it is far more remote a possibility (if it will ever be possible) due to the political and legal realities restricting embryo research, and the uncertain limits of embryo science.

This is even the case for newborns in NIC that, while assisted by technology, must have some ability to exercise their own independent capacity for life; otherwise, they would not be able to be sustained with conventional assistance.^1^

This is also the case in the law of multiple other jurisdictions that recognise birth as a meaningful legal moment (usually the trigger of legal personality).

This is at least the case in English and Welsh law.

Interestingly, an Australian Court of Appeal has recently endorsed a broader approach to determining if a developing human entity is ‘alive’. In *R v Iby*,^18^ Spigelman J observed that ‘the common law “born alive” rule is satisfied by an indicia of independent life. There is no single test of what constitutes “life”’.

Notably, Colgrove did not meaningfully respond to these points of difference I raised in my earlier publication.

I am grateful to Professor Bernard Dickens for raising this point with me in discussion.

Even those who subscribe to a gradualist approach take a moral position based on the assumption that all life is valuable (similar to the sanctity of life approach) but that there are limits on how seriously we should take that value.

Most people have an opinion or an intuition on the subject (eg, I believe that the personhood approach has more merit). However, these positions are not empirically provable.

Especially as I have demonstrated that the gestateling is not ontologically identical to either.

It is important to note that pregnancy is a condition that can be experienced by any person with the reproductive biology that allows them to become pregnant (female) irrespective of gender identity. In this paper, I generally have referred to pregnant persons rather than pregnant women in order to encapsulate this fact. However, here I refer to a choice that women must be allowed to make because the vast majority of people who experience pregnancy identify as women, and this massively impacts on the social experience of pregnant people and the choice to terminate a pregnancy.
